# Impact of comorbidities and use of common medications on cancer and non-cancer specific survival in esophageal carcinoma

**DOI:** 10.1186/s12885-015-1095-2

**Published:** 2015-03-09

**Authors:** Li-Ru He, Wei Qiao, Zhong-Xing Liao, Ritsuko Komaki, Linus Ho, Wayne L Hofstetter, Steven H Lin

**Affiliations:** 1 Departments of Radiation Oncology, The University of Texas MD Anderson Cancer Center, Houston, TX USA; 2 Biostatistics, The University of Texas MD Anderson Cancer Center, Houston, TX USA; 3 Gastrointestinal Medical Oncology, The University of Texas MD Anderson Cancer Center, Houston, TX USA; 4 Thoracic and Cardiovascular Surgery, The University of Texas MD Anderson Cancer Center, Houston, TX USA; 5 Department of Radiation Oncology, Cancer Center, Sun Yat-Sun University, Guangzhou, China

**Keywords:** Esophageal carcinoma, Comorbidity, Medication, Survival

## Abstract

**Background:**

Chronic comorbidities and some of the commonly-used medications are thought to affect cancer patients’ outcomes, but their relative impact on esophageal carcinoma (EC) has not been well studied. The purpose of the study was to identify the chronic comorbidities and/or commonly-used medications that impact EC patient survival.

**Methods:**

A total of 1174 EC patients treated with chemoradiotherapy (CRT) with or without surgery in one institution from 1998 to 2012 were retrospectively included. Seven kinds of frequently occurring chronic comorbidities and 18 types of regularly-taken medications were obtained from medical records. Since it is expected prognostic factors have different effects between surgery patients and non-surgery patients, the impact value of all variables and the corresponding interactions with surgery on survival were evaluated in Cox proportional hazards regression model. Overall mortality, EC-specific mortality and non EC-specific mortality were endpoints.

**Results:**

We found that atrial fibrillation was the only comorbidity that showed a significant impact on non-EC specific survival for all patients (HR 1.72, *P* = 0.03), whereas hypothyroidism was the only comorbidity that was evaluated as an independent predictive factor for overall survival (OS) (HR 0.59, *P* = 0.02) and EC-specific survival (HR 0.62, *P* = 0.05), but this association was seen only in the non-surgical patients. No other medications were found to have a significant impact for OS, EC-specific survival or non-EC specific survival in multivariable analysis.

**Conclusions:**

Our data indicate that certain comorbidities rather than medication use affect EC-specific survival or non EC-specific survival in EC patients treated with CRT with or without surgery. Comorbidity information may better guide individual treatment in EC.

## Background

Concurrent chemoradiotherapy (CRT) followed by surgery is widely accepted as the standard treatment for locally advanced esophageal carcinoma (EC). However, there is still a portion of patients being excluded from this curative combined therapy mainly because of the poor performance status due to comorbidities [1]. Until now, how these common comorbidities influence EC patient survival is known to a limited degree. In a retrospective study of a large Esophagogastric Cancer Registry, postoperative mortality was found to increase in patients of advanced age and with greater comorbidity [2]. By contrast, another report recently revealed that there was no increased risk for mortality in EC patients with diabetes or other common comorbidities selected for surgery [3]. So far, the limited prior studies focused mainly on EC patients treated with surgery and with inconsistent results. Even less is known on how these comorbidities affect clinical outcomes for patients treated without surgery.

For patients with certain comorbidities, the medications used for treating these ailments are inevitably used throughout the treatment course. Recently, the importance of the medication information has attracted more and more attention. Firstly, a key advantage for analyzing medication use is the objectivity and better accuracy in assessing a patient’s underlying health conditions than past medical history documentation. Second, the dose of the medications may provide a better perspective on the severity of the comorbid condition. Third, the use of some medications has already been reported to be associated with the risk and/or therapy response of EC [4-6]. However the degree these drugs affect prognosis of esophageal cancer is not known. Furthermore, the relative impact that comorbid disease has on prognosis as compared to the use of certain medications for the specific ailments is also not well understood.

The purpose of our study was to understand the relative impact that comorbid diseases and medication use have on the patients’ survival. We evaluated how these two factors influenced EC-specific death and non EC-specific death in a large cohort of EC patients treated with CRT with or without surgery.

## Methods

### Patient selection

All patients had histologically proven primary esophageal carcinoma and treated with concurrent CRT with or without esophagectomy. A total of 1174 patients (560 and 614 patients with and without esophagectomy, respectively) treated in our institution from January 1998 to April 2012 were included for this analysis. This study was approved by the institutional review board of The University of Texas MD Anderson Cancer Center and was performed in accordance with the Declaration of Helsinki [7].

### Evaluations and interventions

Staging and restaging was done according to the 6th (2002) edition of the American Joint Committee on Cancer (AJCC) staging manual for esophageal carcinoma. Patients were treated with concurrent CRT with or without induction chemotherapy and following esophagectomy. Radiation was delivered with 3-dimensional conformal radiation (3D-CRT), intensity-modulated radiation (IMRT), or proton beam therapy. The typical radiation dose was 50.4 Gy in 28 fractions. All patients received platin- or taxane-based chemotherapy with fluorouracil. CRT response was evaluated according to the Response Evaluation Criteria in Solid Tumors (RECIST) system at 0–3 months after the completion of CRT. Esophagectomy was approved by the thoracic multidisciplinary group according the re-evaluation after CRT, and was performed 4–8 weeks after CRT completion.

### Data collection

Medical records were reviewed for baseline characteristics, preexisting chronic comorbidities, preexisting regularly-taken medications, treatment modalities, tumor control and patients’ survival outcomes. According to the past medical history record, the preexisting chronic comorbidities including the following 4 most frequently occurring groups: (1) hypertension; (2) cardiovascular disease (coronary artery disease [CAD] and atrial fibrillation [AF] (any types included, intermittent or persistent)); (3) pulmonary disease (chronic obstructive pulmonary disease [COPD] and asthma) and (4) metabolic diseases (diabetes and hypothyroidism). Other medical comorbidities which included less than 2.5% (30) of the patients were not included in the analysis, such as cerebrovascular disease, gout, hyperthyroidism, anemia and prostatic hypertrophy.

In total, 12 kinds of medications used for the above comorbidities were also recorded: (1) anti-hypertensive drugs (angiotensin-converting enzyme inhibitor/angiotensin receptor blockers (ACEi/ARB), beta-blocker, calcium channel antagonist, alpha-1-adrenoceptor blocker and diuretic) (2) cardiovascular drugs (cardiac glycoside and coronary vasodilator), (3) bronchodilators, (4) hypoglycemic agents (insulin, sulfonylureas, biguanide) and (5) levothyroxine. Other antiarrhythmic drugs except beta-blocker and cardiac glycoside, and other hypoglycemic drugs were not included because the frequency was less than 2.5% of the patients.

In addition, 6 kinds of other medications, frequently used by this cohort of the patients, were also included: (1) antacids, (2) non-steroidal anti-inflammatory drugs (NSAIDs), (3) antihyperlipidemics (statins and other lipid-regulating agents), (4) antithrombotics and (5) antidepressants. Since all the patients recorded as having hypothyroidism also regularly took levothyroxine, hypothyroidism/levothyroxine was considered one variable in analysis.

### Outcome definition

Local/regional failure was defined as the persistence or recurrence of the primary tumor and regional lymph nodes, while distant failure was defined as metastasis to any site beyond the primary tumor and regional lymph nodes. OS, EC-specific survival and non EC-specific survival were defined as the time from the end of CRT to any cause of death, either due to esophageal carcinoma or any cause of death other than esophageal carcinoma, respectively. Since the date record of CRT end is missing for one patient treated in 1998, leaving 1173 patients for survival analysis.

### Statistical analysis

The distribution of each categorical variable was summarized in terms of its frequencies and percentages. Fisher’s exact texts were used to assess measures of association in frequency tables. Survival curves were obtained with the Kaplan-Meier method and compared with log-rank tests. The Cox proportional hazards regression model was used to evaluate the ability of patient prognostic variables or surgery effect to predict survival. Since receipt of surgery has been recognized as a major prognostic factor for loco-regional EC and it is expected that the prognostic factors would have different impacts on survival between surgery patients and non-surgery patients, the interaction term of each prognostic factor and surgery is included for each variable in the univariable analysis. The variables with either potentially significant main effect or the interaction term (*P* < 0.10) were selected and included in the multivariable mode for OS, EC-specific survival and non EC-specific survival. A *P* value less than 0.05 was considered statistically significant in multivariable analysis. For each significant interaction term in the multivariate model, it indicates that the corresponding variable affect survival differently in surgery and non-surgery patient. Hence, the hazard rate (HR) for death, 95% confidence interval [CI] and *P* value of the variable were further calculated for surgery and non-surgery patients respectively. All computations were carried out in SAS version 9.3. (SAS Institute, Cary, NC) and all statistical tests were 2-sided.

## Results

### Patient characteristics, comorbidities and medications

Baseline characteristics of the 1174 EC patients in our cohort are listed in Table 1. The frequencies of the major comorbidities and medications are presented in Table 2. The most prevalent comorbidity was hypertension, followed by diabetes, CAD, hypothyroidism, COPD and asthma. Antacid, NSAIDS, statins, ACEi/ARB and beta-blocker were the top five frequently used medications.

**Table 1:** **Patient and tumor characteristics**

Characteristics	Value or No. of patients (%)
Age at diagnosis (years)	
Median (Range)	64 (20-91)
Gender	
Female	182(15.5)
Male	992(84.5)
Race	
White	1028(87.6)
Non-white	146(12.4)
BMI	
≤25	285(24.3)
>25	704(60.0)
Not applicable	185(15.7)
KPS	
**≤** 70	117(10.0)
80-100	1057(90.0)
Heavy alcohol use history	250(21.3)
Smoking at diagnosis	248 (21.1)
No	924(78.7)
Yes	250(21.3)
Second malignancy	186(15.8)
Tumor location	
Proximal/ Middle	159(13.5)
Distal	1015(86.5)
Tumor histology	
ADE	914(77.9)
SCC	237(20.2)
Others	23(1.9)
Tumor differentiation	
Well/ Moderate	517(44.0)
Poor	644(54.9)
Not applicable	13(1.1)
Tumor length (cm)	
Median(Range)	5(0.4-20.0)
Clinical stage	
I-II	432(36.8)
III-IV	714(60.8)
Not applicable	28(2.4)
Induction chemotherapy	468 (40.0)
Radiation modality	
3DCRT	469(39.9)
IMRT/Proton	705(60.1)
Surgery	560(47.7)

KPS: Karnofsky performance scores; BMI: body mass index; ADE: adenocarcinoma; SCC: squamous cell carcinoma; 3DCRT: 3-dimensional conformal radiation; IMRT: intensity-modulated radiation.

**Table 2:** **Univariate survival analysis of comorbidities, medications and their interactions with surgery**

		Overall survival		EC-specific survival		Non-EC specific survival	
Variables	No. (%)	HR(95% CI)^1^	*P* ^1^	HR(95% CI)^1^	*P* ^1^	HR(95% CI)^1^	*P* ^1^
Hypertension	620(52.8)	0.92(0.76-1.11)	0.38	0.92(0.73-1.16)	0.47	0.91(0.66-1.26)	0.57
Interaction with surgery		1.18(0.88-1.60)	0.27	1.00(0.69-1.45)	1.00	1.67(0.99-2.81)	0.05
CAD	184(15.7)	1.00(0.80-1.25)	0.99	0.88(0.66-1.16)	0.36	1.26(0.86-1.84)	0.24
Interaction with surgery		1.05(0.67-1.66)	0.83	0.94(0.51-1.70)	0.83	1.29(0.63-2.64)	0.49
AF	63(5.4)	1.23(0.89-1.71)	0.22	0.84(0.53-1.34)	0.48	2.19(1.36-3.51)	<0.01
Interaction with surgery		0.63(0.28-1.44)	0.28	0.57(0.17-1.95)	0.37	0.68(0.22-2.05)	0.49
COPD	65(5.5)	1.14(0.80-1.62)	0.47	0.69(0.40-1.18)	0.17	2.22(1.37-3.59)	<0.01
Interaction with surgery		0.66(0.29-1.52)	0.33	0.66(0.19-2.31)	0.51	0.68(0.22-2.08)	0.50
Asthma	36(3.1)	1.09(0.68-1.75)	0.71	0.79(0.41-1.53)	0.48	1.81(0.92-3.56)	0.09
Interaction with surgery		1.38(0.59-3.22)	0.46	1.80(0.59-5.46)	0.30	0.93(0.25-3.56)	0.92
Diabetes	193(16.4)	1.10(0.87-1.40)	0.43	1.13(0.85-1.51)	0.39	1.04(0.68-1.61)	0.84
Interaction with surgery		0.98(0.64-1.50)	0.92	0.72(0.42-1.26)	0.25	1.59(0.81-3.15)	0.18
Hypothyroidism/levothyroxine	102(8.7)	0.59(0.42-0.83)	<0.01	0.52(0.33-0.80)	<0.01	0.74(0.43-1.29)	0.29
Interaction with surgery		2.26(1.32-3.86)	<0.01	2.27(1.14-4.52)	0.02	2.21(0.94-5.21)	0.07
ACEi/ARB	350(29.8)	0.99(0.81-1.21)	0.95	0.94(0.73-1.20)	0.60	1.10(0.78-1.56)	0.58
Interaction with surgery		1.06(0.76-1.47)	0.72	0.97(0.64-1.46)	0.87	1.28(0.74-2.22)	0.38
beta-Blocker	217(18.5)	0.95(0.76-1.18)	0.65	1.05(0.81-1.36)	0.73	0.74(0.49-1.12)	0.16
Interaction with surgery		1.07(0.71-1.61)	0.73	0.71(0.42-1.21)	0.21	2.29(1.18-4.43)	0.01
Calcium antagonist	172(14.7)	1.01(0.79-1.30)	0.92	1.05(0.78-1.42)	0.73	0.94(0.60-1.48)	0.79
Interaction with surgery		1.24(0.82-1.86)	0.31	1.06(0.63-1.77)	0.82	1.63(0.82-3.23)	0.16
alpha-1-Adrenoceptor blocker	105(8.9)	1.19(0.90-1.58)	0.23	1.08(0.76-1.55)	0.67	1.45(0.90-2.32)	0.12
Interaction with surgery		1.12(0.67-1.87)	0.66	1.05(0.54-2.04)	0.89	1.20(0.54-2.70)	0.65
Diuretic	200(17.0)	1.01(0.80-1.26)	0.96	0.93(0.7-1.24)	0.63	1.13(0.77-1.66)	0.53
Interaction with surgery		1.18(0.79-1.77)	0.42	1.27(0.77-2.08)	0.35	1.07(0.53-2.16)	0.85
Cardiac glycoside	48(4.1)	1.04(0.72-1.51)	0.83	0.67(0.38-1.17)	0.16	1.83(1.11-3.04)	0.02
Interaction with surgery		2.09(0.94-4.62)	0.07	2.41(0.77-7.51)	0.13	1.82(0.59-5.60)	0.30
Coronary vasodilator	33(2.8)	0.61(0.36-1.04)	0.07	0.67(0.35-1.25)	0.21	0.50(0.19-1.36)	0.18
Interaction with surgery		1.66(0.66-4.15)	0.28	0.69(0.15-3.18)	0.63	3.89(1.02-14.88)	0.05
Bronchodilator	33(2.8)	1.34(0.84-2.15)	0.22	0.53(0.22-1.27)	0.15	3.35(1.89-5.93)	<0.01
Interaction with surgery		0.73(0.27-1.98)	0.53	2.05(0.54-7.74)	0.29	0.21(0.03-1.62)	0.13
Insulin	37(3.2)	0.81(0.47-1.37)	0.43	0.66(0.32-1.32)	0.24	1.17(0.52-2.65)	0.71
Interaction with surgery		1.87(0.67-5.25)	0.23	1.27(0.27-6.07)	0.76	2.76(0.67-11.31)	0.16
Sulfonylurea	75(6.4)	1.34(0.95-1.88)	0.09	1.53(1.04-2.26)	0.03	0.94(0.46-1.93)	0.87
Interaction with surgery		0.71(0.37-1.37)	0.31	0.35(0.13-0.92)	0.03	2.02(0.73-5.59)	0.18
Biguanide	104(8.9)	0.99(0.70-1.40)	0.97	1.11(0.75-1.65)	0.60	0.74(0.36-1.51)	0.41
Interaction with surgery		0.85(0.48-1.49)	0.57	0.62(0.31-1.26)	0.19	1.62(0.60-4.35)	0.34
Antacid	657(56.0)	0.74(0.62-0.89)	<0.01	0.79(0.63-0.99)	0.04	0.67(0.48-0.92)	0.01
Interaction with surgery		1.25(0.92-1.69)	0.15	1.21(0.84-1.76)	0.31	1.30(0.77-2.19)	0.30
NSAIDs	507(43.2)	1.10(0.92-1.33)	0.30	1.06(0.84-1.33)	0.63	1.19(0.86-1.64)	0.30
Interaction with surgery		0.90(0.66-1.22)	0.50	0.89(0.61-1.29)	0.54	0.94(0.55-1.6)	0.82
Statins	400(34.1)	0.84(0.69-1.02)	0.08	0.81(0.64-1.04)	0.09	0.87(0.62-1.23)	0.43
Interaction with surgery		1.19(0.86-1.64)	0.30	1.05(0.70-1.56)	0.81	1.53(0.89-2.66)	0.13
**Variables**	**NO. (%)**	**HR(95%CI)** ^**1**^	***P*** ^**1**^	**HR(95%CI)** ^**1**^	***P*** ^**1**^	**HR(95%CI)** ^**1**^	***P*** ^**1**^
Other lipid-regulating agents	60(5.1)	0.88(0.55-1.41)	0.60	1.08(0.64-1.82)	0.77	0.46(0.15-1.45)	0.19
Interaction with surgery		0.75(0.36-1.56)	0.45	0.41(0.16-1.07)	0.07	2.53(0.64-10.06)	0.19
Antithrombotic	119(10.1)	0.99(0.75-1.30)	0.94	0.86(0.61-1.22)	0.40	1.29(0.83-2.00)	0.25
Interaction with surgery		1.03(0.59-1.80)	0.93	0.91(0.43-1.93)	0.80	1.19(0.51-2.78)	0.68
Antidepressant	215(18.3)	0.99(0.79-1.24)	0.92	0.98(0.74-1.30)	0.91	1.01(0.67-1.50)	0.97
Interaction with surgery		1.06(0.72-1.57)	0.76	0.89(0.55-1.47)	0.66	1.43(0.75-2.73)	0.27

^1^Other factors analyzed in univariate analysis include: age, sex, race, BMI, heavy alcohol use history, smoking at diagnosis, second malignancy, Karnofsky performance scores, tumor histology, tumor location, tumor differentiation, clinical stage, induction chemotherapy, radiation modality and their interactions with surgery.

EC: esophageal carcinoma; HR: hazard rate; CI: confidence interval; CAD: coronary artery disease; AF: atrial fibrillation; COPD: chronic obstructive pulmonary disease; ACEi: angiotensin-converting enzyme inhibitor; ARB: angiotensin receptor blocker; NSAIDs: non-steroidal anti-inflammatory drugs.

### Impact of comorbidities and medications on outcomes

**Figure 1:**
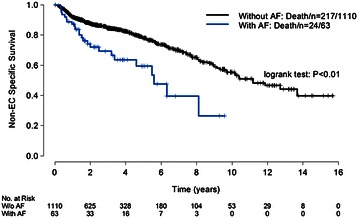
**Non-esophageal carcinoma specific survival for patients with and without atrial fibrillation.**

The median follow-up for the whole cohort was 25 months (3 to 186 months) with a 5y-OS of 38%. Besides the comorbidities and medications, the impact value of age, sex, race, body mass index (BMI), heavy alcohol use history, smoke at diagnosis, second malignancy, Karnofsky performance scores, tumor histology, tumor location, tumor differentiation, clinical stage, induction chemotherapy, radiation modality and their interactions with surgery were all tested in univariate analysis. Other factors which showed a significant impact on OS, EC-specific survival or non-EC specific survival in univariate analysis were listed in the footnote of Table 2. All the parameters included in the multivariate analysis were listed in the footnote of Table 3. After adjusting for patients’ baseline characteristics, AF was the only comorbidity that showed a significant impact on non-EC specific survival in both univariable (Table 2, Figure 1) and multivariable analysis (Table 3). For OS and EC-specific survival, hypothyroidism/levothyroxine was also the only significant factor in both univariable and multivariable analysis, with a significant interaction with surgery. It had a significant impact on OS (HR 0.59, 95% CI 0.38–0.93, *P* = 0.02) and EC-specific survival (HR 0.62, 95% CI 0.38–1.01, *P* = 0.05) for non-surgery patients but not for the surgery patients.

**Table 3:** **Multivariate survival analysis of comorbidities, medications and their interactions with surgery**

	Overall survival		EC-specific survival		Non-EC specific survival	
Variables	HR(95% CI)^1^	*P* ^1^	HR(95% CI)^2^	*P* ^2^	HR(95% CI)^3^	*P* ^3^
Hypertension	-	-	-	-	0.94(0.66-1.33)	0.71
Interaction with surgery	-	-	-	-	1.36(0.77-2.41)	0.29
AF	-	-	-	-	1.72(1.07-2.77)	0.03
Interaction with surgery	-	-	-	-	-	-
COPD	-	-	-	-	1.43(0.88-2.38)	0.15
Interaction with surgery	-	-	-	-	-	-
Asthma	-	-	-	-	1.42(0.75-2.70)	0.28
Interaction with surgery	-	-	-	-	-	-
Hypothyroidism/ levothyroxine	0.59(0.38-0.93)	0.02	0.62(0.38-1.01)	0.05	0.78(0.44-1.37)	0.39
Interaction with surgery	2.04(1.09-3.83)	0.03	2.20(1.03-4.69)	0.04	1.50(0.61-3.69)	0.38
beta-Blocker	-	-	-	-	0.70(0.45-1.07)	0.10
Interaction with surgery	-	-	-	-	2.26(1.09-4.68)	0.03
Cardiac glycoside	1.50(0.92-2.44)	0.10	-	-	1.31(0.78-2.20)	0.30
Interaction with surgery	1.19(0.48-2.96)	0.71	-	-	-	-
Coronary vasodilator	0.93(0.54-1.61)	0.80	-	-	0.45(0.16-1.28)	0.13
Interaction with surgery	-	-	-	-	2.92(0.72-11.97)	0.13
Bronchodilator	-	-	-	-	1.89(0.98-3.62)	0.06
Interaction with surgery	-	-	-	-	-	-
Sulfonylurea	1.15(0.81-1.65)	0.43	1.28(0.79-2.07)	0.32	-	-
Interaction with surgery	-	-	0.49(0.18-1.36)	0.17	-	-
Antacid	0.94(0.79-1.12)	0.47	0.92(0.75-1.13)	0.41	0.80(0.61-1.05)	0.11
Interaction with surgery	-	-	-	-	-	-
Statin	0.92(0.76-1.11)	0.38	0.87(0.70-1.07)	0.19	-	-
Interaction with surgery	-	-	-	-	-	-
Other lipid-regulating agents	-	-	0.98(0.56-1.71)	0.94	-	-
Interaction with surgery	-	-	0.66(0.24-1.77)	0.40	-	-

^1^Adjusted for the interactions of age, race, tumor histology and tumor location with surgery, BMI, smoking at diagnosis, Karnofsky performance scores, tumor location, clinical stage, radiation modality and surgery for overall survival.

^2^Adjusted for the interactions of race, age, tumor histology and tumor location with surgery, sex, race, age, smoking at diagnosis, tumor histology, tumor length, tumor differentiation, clinical stage, radiation modality and surgery for EC-specific death free survival.

^3^Adjusted for the interactions of tumor histology and tumor location with surgery, age, Karnofsky performance scores, tumor histology, induction chemotherapy, radiation modality and surgery for non-EC specific death free survival.

EC: esophageal carcinoma; HR: hazard rate; CI: confidence interval; AF: atrial fibrillation; COPD: chronic obstructive pulmonary disease.

**Figure 2:**
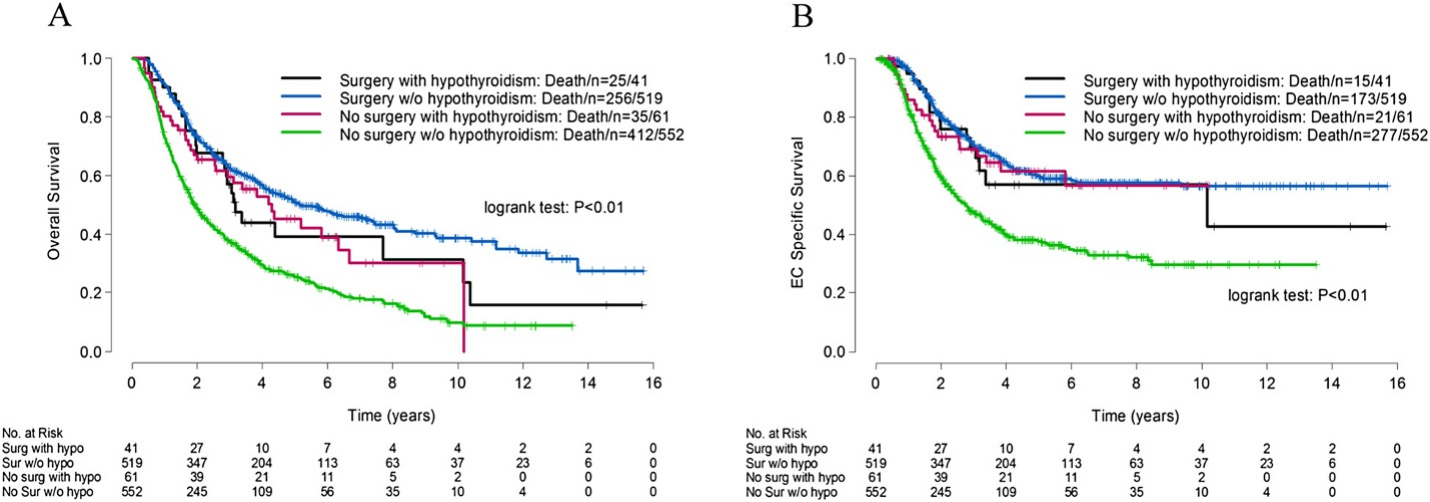
**Survival stratified by hypothyroidism and surgery status for patients with esophageal carcinoma. A**: Overall survival, **B**: Esophageal carcinoma-specific survival.

To better interpret the significant interaction of hypothyroidism/levothyroxine and surgery for OS and EC-specific survival, the survival curves stratified by hypothyroidism/levothyroxine and surgery were further presented in Figure 2A and B. The 5 year OS (45% vs. 25%, *P* = 0.003) and EC-specific survival (62% vs. 38%, *P* = 0.004) for patients with hypothyroidism/levothyroxine was significant higher than those without hypothyroidism/levothyroxine for non-surgery patients but not for surgery patients (*P* > 0.05).

### Characteristics difference between patients with/without AF and hypothyroidism/levothyroxine

Since AF and hypothyroidism/levothyroxine were found to significantly impact patients’ survival, we compared the difference of the clinico-pathologic characteristics between patients with and without AF (Table 4) and hypothyroidism/levothyroxine (Table 5), respectively. There were more patients who are older than 64 yrs (*P* < 0.01), had no surgery (*P* < 0.01), treated with IMRT/proton therapy (*P* < 0.01), without a complete CRT response (*P* = 0.02) and had a lower distant failure rate (*P* = 0.01) in the AF group than in non-AF group. No difference was observed on other clinico-pathologic characteristics between the two groups (Table 4).

**Table 4:** **Characteristics of esophageal carcinoma patients with or without Atrial fibrillation**

		Atrial fibrillation		*P* ^1^
Variables		No	Yes	
Sex	Female	174(15.7%)	8(12.7%)	0.72
	Male	937(84.3%)	55(87.3%)	
Race	non-White	143(12.9%)	3(4.8%)	0.07
	White	968(87.1%)	60(95.2%)	
BMI	≤25	271(29.1%)	14(24.1%)	0.45
	>25	660(70.9%)	44(75.9%)	
Age (years)	≤64	601(54.1%)	12(19%)	<0.01
	>64	510(45.9%)	51(81%)	
Heavy alcohol use history	No	868(78.3%)	54(85.7%)	0.21
	Yes	241(21.7%)	9(14.3%)	
Smoke at diagnosis	No	869(78.5%)	53(84.1%)	0.34
	Yes	238(21.5%)	10(15.9%)	
KPS	≤70	107(9.6%)	10(15.9%)	0.13
	>70	1004(90.4%)	53(84.1%)	
Tumor histology	ADC	864(79.2%)	50(79.4%)	1.00
	SCC	227(20.8%)	13(20.6%)	
Tumor location	Upper/middle	152(13.7%)	7(11.1%)	0.71
	Low	959(86.3%)	56(88.9%)	
Tumor differentiation	Well/moderate	487(44.4%)	30(47.6%)	0.70
	Poor	611(55.6%)	33(52.4%)	
Tumor length (cm)	≤5	564(57.7%)	35(59.3%)	0.89
	>5	414(42.3%)	24(40.7%)	
Clinical stage	I-II	403(37.2%)	28(44.4%)	0.28
	III-IV	680(62.8%)	35(55.6%)	
Surgery	No	567(51%)	47(74.6%)	<0.01
	Yes	544(49%)	16(25.4%)	
Radiation modality	3DCRT	456(41%)	13(20.6%)	<0.01
	IMRT/proton	655(59%)	50(79.4%)	
Induction chemotherapy	No	662(59.6%)	44(69.8%)	0.11
	Yes	449(40.4%)	19(30.2%)	
CRT complete response	No	658(62.3%)	47(77%)	0.02
	Yes	399(37.7%)	14(23%)	
Chemotherapy after relapse	No	948 (85.3%)	56 (88.9%)	0.58
	Yes	163 (14.7%)	7 (11.1%)	
Local/regional failure rate	No	298(30.5%)	17(33.3%)	0.64
	Yes	678(69.5%)	34(66.7%)	
Distant failure rate	No	556(54.9%)	38(73.1%)	0.01
	Yes	456(45.1%)	14(26.9%)	

^1^Fisher exact test.

KPS: Karnofsky performance scores; BMI: body mass index; ADC: adenocarcinoma; SCC: squamous cell carcinoma; 3DCRT: 3-dimensional conformal radiation; IMRT: intensity-modulated radiation: CRT: chemoradiotherapy.

**Table 5:** **Characteristics of esophageal carcinoma patients with or without hypothyroidism/levothyroxine**

		Hypothyroidism/Levothyroxine		*P* ^1^
Variables		No	Yes	
Sex	Female	148(13.8%)	34(33.3%)	<0.01
	Male	924(86.2%)	68(66.7%)	
Race	non-White	131(12.2%)	15(14.7%)	0.44
	White	941(87.8%)	87(85.3%)	
BMI	≤25	262(29.1%)	23(25.8%)	0.62
	>25	638(70.9%)	66(74.2%)	
Age (years)	≤64	569(53.1%)	44(43.1%)	0.06
	>64	503(46.9%)	58(56.9%)	
Heavy alcohol use history	No	839(78.4%)	83(81.4%)	0.53
	Yes	231(21.6%)	19(18.6%)	
Smoke at diagnosis	No	830(77.6%)	92(91.1%)	<0.01
	Yes	239(22.4%)	9(8.9%)	
KPS	≤70	104(9.7%)	13(12.7%)	0.30
	>70	968(90.3%)	89(87.3%)	
AF	No	1013(94.5%)	98(96.1%)	0.65
	Yes	59(5.5%)	4(3.9%)	
Tumor histology	ADC	841(79.9%)	73(71.6%)	0.05
	SCC	211(20.1%)	29(28.4%)	
Tumor location	Upper/middle	140(13.1%)	19(18.6%)	0.13
	Low	932(86.9%)	83(81.4%)	
Tumor differentiation	Well/moderate	477(45.0%)	40(39.6%)	0.35
	Poor	583(55.0%)	61(60.4%)	
Tumor length (cm)	≤5	537(56.9%)	62(66.7%)	0.08
	>5	407(43.1%)	31(33.3%)	
Clinical stage	I-II	383(36.6%)	48(48.5%)	0.02
	III-IV	664(63.4%)	51(51.5%)	
Surgery	No	553(51.6%)	64(59.8%)	0.12
	Yes	519(48.4%)	41(40.2%)	
Radiation modality	3DCRT	430(40.1%)	39(38.2%)	0.75
	IMRT/proton	642(59.9%)	63(61.8%)	
Induction chemotherapy	No	638(59.5%)	68(66.7%)	0.17
	Yes	434(40.5%)	34(33.3%)	
CRT complete response	No	651(63.5%)	54(58.1%)	0.31
	Yes	374(36.5%)	39(41.9%)	
Chemotherapy after relapse	No	914(85.3%)	90(88.2%)	0.47
	Yes	158(14.7%)	12(11.8%)	
Local/regional failure rate	No	290(31.0%)	25(26.9%)	0.48
	Yes	644(69.0%)	68(73.1%)	
Distant failure rate	No	532(55.0%)	62(64.6%)	0.08
	Yes	436(45.0%)	34(35.4%)	

^1^Fisher exact test.

KPS: Karnofsky performance scores; BMI: body mass index; ADC: adenocarcinoma; SCC: squamous cell carcinoma; AF: atrial fibrillation; 3DCRT: 3-dimensional conformal radiation; IMRT: intensity-modulated radiation: CRT: chemoradiotherapy.

There were more patients who are female (*P* < 0.01), without smoke at diagnosis (*P* < 0.01), with squamous cell carcinoma (SCC) histology (*P* = 0.05) and earlier clinical stage (*P* = 0.02) in the hypothyroidism/levothyroxine group than in non-hypothyroidism/levothyroxine group. No difference was observed on other clinico-pathologic characteristics between the two groups (Table 5).

## Discussion

In our retrospective study, by simultaneously analyzing the impact value of 7 kinds of frequently occurring comorbidities and 18 types of regularly-taken medications on EC patient survival, we identified that certain comorbidities (hypothyroidism and AF) but not specific medications that affected EC-specific survival or non-EC specific survival in a large EC cohort treated with CRT with or without surgery.

It is generally recognized that comorbidities may affect patients’ prognosis mainly by impacting the non-cancer specific survival [1]. In addition, patients with comorbidities are more likely to experience severe treatment toxicities and even treatment related death [8]. For example, AF, which remains one of the most frequent complications after esophagectomy, has been reported to be associated with the pre-existing AF and increased postoperative mortality by several studies [9,10]. In our study, we found that AF was an adverse prognostic factor on non-EC specific survival for all CRT treated patients regardless of whether they received surgery or not. Considering the significant impact of AF on the prognosis of EC, an improved management of pre-existing AF in EC patients before and during cancer treatments should be recommended.

Interestingly, preexisting hypothyroidism was a significant protective factor for OS in non-surgical patients, possibly by affecting EC-specific death, since it showed no impact on non-EC specific survival in our analysis. Although the impact of hypothyroidism and human cancer has been a controversial issue [11,12], some recent data suggests that it is associated with a good prognosis of certain human cancers (head and neck, lung and renal cancers) [12-14]. Our study is the first to make this association for EC. We also found that patients with hypothyroidism tended to have earlier clinical stage disease than euthyroid patients, which was also observed for breast cancer patients [15]. The underlying mechanisms that have been proposed for the role of hypothyroidism in cancer are mainly through interfering the process of cell proliferation and apoptosis, since hypothyroidism is characterized by reduced production of thyroid hormone [16]. In animal models, thyroid hormone can stimulate tumor growth and metastasis, whereas hypothyroidism shows the opposite effects [17,18]. While to date, there is no specific study determining the mechanism by which hypothyroidism affect the prognosis of EC. It is unclear why the survival benefit of hypothyroidism was not seen for surgical patients. This observation will need confirmation in future studies.

There have been a number of reports showing that certain medications have an impact on EC. Biguanide (metformin), statins and NSAIDs (aspirin) have been reported to be associated with a clinically reduced EC incidence [6,19] and have an anti-tumor effect in EC cells [20-22]. Recently, a retrospective study showed that metformin use is associated with an increased CRT response in esophageal adenocarcinoma, but no benefit of metformin was observed for OS [4]. In our analysis, we could not identify a single medication effect on patient survival in EC. Although the survival benefit of certain drugs has been reported in some other human cancers [23,24], the recognized heterogeneity among the various studies [25] and the survival influence of certain drugs could be cancer-specific. To date, there are not reports that support the survival influence of any medications on EC patients.

The limitations of our study relate to the retrospective collection of comorbid information from the medical records elicited from physicians’ clinical evaluations, which may underestimate the existence of certain comorbidities if they were not asked or were not willingly provided by the patients. Second, it is important to note that the prevalence of certain comorbidities and medications can affect the statistical power to detect their impact on patient survival. Thus, the lack of the statistical significance for a certain variable with low prevalence should be interpreted with caution. Third, although our data does corroborate previously published studies supporting the protective role of hypothyroidism in certain types of human cancers, we can’t exclude the influence of levothyroxine on EC prognosis in our study, as all the patients with hypothyroidism also took levothyroxine. In addition, there is also a possibility that patients may take levothyroxine due to reasons other than hypothyroidism. Further studies are needed to better clarify the roles of hypothyroidism and levothyroxine on EC prognosis in different cohort of EC patients.

## Conclusion

In conclusion, despite the growing evidence that some medications and/or their underlying comorbidities predict patients’ prognosis in some human cancers, certain comorbidities (hypothyroidism and AF) rather than commonly-used medications affect patient survival in EC patients treated with CRT with or without surgery. Comorbidity information should be taken into consideration when individualized treatment decisions are made for EC patients.

## Abbreviations

EC: Esophageal carcinoma
CRT: Chemoradiotherapy
OS: Overall survival
3DCRT: 3-dimensional conformal radiation
IMRT: Intensity-modulated radiation
CAD: Coronary artery disease
AF: Atrial fibrillation
COPD: Chronic obstructive pulmonary disease
NSAIDs: Non-steroidal anti-inflammatory drugs
ACEi: Angiotensin-converting enzyme inhibitor
ARB: Angiotensin receptor blockers
HR: Hazard rate
CI: 95% confidence interval
SCC: Squamous cell carcinoma
ADE: Adenocarcinoma
T_3_: Triiodothyronine
TR: Thyroid hormone nuclear receptors
KPS: Karnofsky performance scores
BMI: Body mass index
